# Deleterious effects of nervous system in the offspring following maternal SARS-CoV-2 infection during the COVID-19 pandemic

**DOI:** 10.1038/s41398-022-01985-z

**Published:** 2022-06-06

**Authors:** Ruting Wang, Zifeng Wu, Chaoli Huang, Kenji Hashimoto, Ling Yang, Chun Yang

**Affiliations:** 1 Department of Cardiology, The Third Affiliated Hospital of Soochow University, Changzhou, 213003 China; 2 Department of Anesthesiology and Perioperative Medicine, The First Affiliated Hospital of Nanjing Medical University, Nanjing, 210029 China; 3 Division of Clinical Neuroscience, Chiba University Center for Forensic Mental Health, Chiba, 260-8670 Japan

**Keywords:** Physiology, Depression

## Abstract

During the Coronavirus disease 2019 (COVID-19) pandemic, severe acute respiratory syndrome coronavirus 2 (SARS-CoV-2) is universally susceptible to all types of populations. In addition to the elderly and children becoming the groups of great concern, pregnant women carrying new lives need to be even more alert to SARS-CoV-2 infection. Studies have shown that pregnant women infected with SARS-CoV-2 can lead to brain damage and post-birth psychiatric disorders in offspring. It has been widely recognized that SARS-CoV-2 can affect the development of the fetal nervous system directly or indirectly. Pregnant women are recommended to mitigate the effects of COVID-19 on the fetus through vaccination, nutritional supplements, and psychological support. This review summarizes the possible mechanisms of the nervous system effects of SARS-CoV-2 infection on their offspring during the pregnancy and analyzes the available prophylactic and treatment strategies to improve the prognosis of fetal-related neuropsychiatric diseases after birth.

## Introduction

Coronavirus disease 2019 (COVID-19) is a highly infectious disease caused by the novel coronavirus, severe acute respiratory syndrome coronavirus 2 (SARS-CoV-2). The World Health Organization (WHO) declared COVID-19 as a global pandemic in March 2020. It has since been called “the most critical global health disaster of the century and the greatest challenge facing humanity since World War II” [1, 2]. COVID-19 has spread rapidly across the globe, posing enormous health, economic and social challenges to humanity. As a respiratory infectious disease, SARS-CoV-2 is transmitted mainly through droplets, respiratory secretions, and direct contact [3]. However, recent studies have shown that SARS-CoV-2 can also affect the health of the next generation through vertical transmission from mother to child [4, 5]. Although COVID-19 is considered as a respiratory disease with the main clinical manifestations of fever, cough, and malaise, SARS-CoV-2 still causes damage to other organs, including the central nervous system (CNS) with symptoms such as dizziness, headache, and impaired consciousness [6]. Current human autopsy studies have determined that patients who died from COVID-19 had detectable viral RNA transcription products in brain tissue, viral proteins in endothelial cells of the olfactory bulb, and genetic sequencing of the cerebrospinal fluid showed the presence of SARS-CoV-2, suggesting that SARS-CoV-2 has the ability to invade the nervous system [7–9]. During the COVID-19 pandemic, the number of pregnant women infected with SARS-CoV-2 worldwide has been steadily increasing, and we have to consider whether maternal infection may have adverse effects on their offspring. The fetal nervous system is in a state of development from the third week of gestation and this process continues until adulthood [10]. During this period, any deviation can lead to nervous system developmental defects and cognitive impairment [11]. Considering the risk of maternal infection on neuropsychiatric disorders in the offspring, this review summarizes the mechanisms of nervous system effects of SARS-CoV-2 infection in pregnant women on their offspring and analyzes feasible treatment modalities.

## Mechanisms

### Maternal immune activation (MIA)

Since the COVID-19 epidemic, several studies have reported that the clinical symptoms and laboratory findings of patients with combined SARS-CoV-2 infection in pregnancy are consistent with those of the general population, with patients showing increased C-reactive protein (CRP) and decreased lymphocytes in their hemogram [12]. Tanacan et al. found that pregnant women with COVID-19 had significantly increased pregnancy complications and inflammatory markers [13]. In addition, the inflammatory cytokines interferon (IFN)-γ and interleukin (IL)-6 were significantly increased, while IL-10 and IL-17 were decreased [13]. Since the fetus is a semi-allograft to the maternal body, pregnancy is also a specific immune adaptation process [14]. On the one hand, increased production of inflammatory cytokines such as IL-4 and IL-10 provides a suitable microenvironment of immunologic tolerance. On the other hand, altered expression of inflammatory cytokines such as IL-1 and tumor necrosis factor (TNF)-α is associated with increased pregnancy complications, such as miscarriage, and preterm delivery [15, 16]. Thus, homeostasis of inflammatory cytokines is essential to a healthy pregnancy, while elevated levels of inflammatory cytokines suggest a state of MIA.

#### Fetal nervous system injury

Maternal trophoblasts, specific natural killer cells, and meconium leukocytes secrete IFN-γ during the pregnancy and are involved in the differentiation of meconium natural killer cells, placenta formation, and meconium maintenance [17]. However, congenital infection causes miscarriage and reduced IFN-γ levels. IFN-γ levels have been reported to be lower in patients with severe COVID-19 infection than in healthy pregnant women and in patients with mild to moderate infection [18]. Similarly, a significant increase in IL-6 levels is observed in patients with severe COVID-19 [19]. Excessive production of IL-6 is associated with adverse pregnancy outcomes, such as preterm birth, premature rupture of membranes, and chorioamnionitis [20]. On the contrary, IL-10 is involved in the immune tolerance process during the pregnancy due to its anti-inflammatory effects and is mainly produced by placental chorionic trophoblasts, uterine natural killer cells, and metaphase mononuclear cells [21]. The decreased IL-10 levels in pregnant women with COVID-19 may be a factor in the impaired immune tolerance in this population and is associated with miscarriage [22]. Adverse pregnancy outcomes such as preterm birth, premature rupture of membranes, and chorioamnionitis can cause neonatal brain damage and even lifelong nervous system disorders [23]. In addition, increased secretion of inflammatory cytokines such as IL-8, IL-1β, and CRP, were found to be associated with microcephaly, ventricular enlargement, and low intelligent quotient [24], suggesting that inflammatory cytokines can cross the placenta and blood-brain barrier (BBB) and directly affect fetal neurodevelopment.

#### Vulnerability to psychiatric disorders in offspring

There is a study that reported that pregnant women during the influenza pandemics had a high incidence of psychiatric disorders in their offspring, including schizophrenia, autism spectrum disorder (ASD), and attention-deficit hyperactivity disorder (ADHD) [25]. Similar results have been obtained in animal experiments, where an animal model of maternal immune activation was constructed by administering polyinosinic- polycytidylic acid [Poly (I:C)] to pregnant mice, and it was found that exposure of mice on days 10–12 of embryonic life resulted in damage to the developing basal ganglia and offspring with defects in prepulse inhibition and latent inhibition that are similar to those found in ASD and schizophrenia individuals [26]. A study of Chinese mothers during the COVID-19 epidemic showed that most mothers were infected with SARS-CoV-2 around the third trimester, and their offspring had reduced motor, communication, and social performance compared to normal levels at 3 months of age [27]. The occurrence of infection in mothers during the first trimester may be a risk factor for ASD and schizophrenia in their offspring [28]. Given that IL-6 has the potential to alter cognitive behavior in offspring [29] and that elevated levels of IL-8, TNF-α, and CRP are associated with an increased risk of schizophrenia in offspring [30, 31], the effects of immune activation responses induced by maternal SARS-CoV-2 infection on fetal brain development may make the offspring more susceptible to neuropsychiatric disorders.

### Direct effects of SARS-CoV-2

Past epidemiological studies have shown that a large number of viral infections during maternal pregnancy can lead to abnormalities in the nervous system in the fetus [32]. Although studies show that the probability of mother-to-child transmission of COVID-19 is extremely low, the possibility of vertical transmission has been reported in several cases [5, 33]. In a study from Wuhan, China, a primigravida was diagnosed with SARS-CoV-2 infection at 34 weeks of gestation and delivered a baby by cesarean section in a negative pressure isolation room at 38 weeks of gestation, during which she wore an N95 mask without contact with the newborn [4]. However, the levels of IgM and IgG antibodies were significantly elevated 2 h after birth, suggesting the possibility of intrauterine infection. It is known that IgM antibodies cannot pass through the placenta, while IgG antibodies can be transmitted through the placenta, and IgM antibodies usually appear 3–7 days after infection, suggesting that SARS-CoV-2 may cause indirect infection of the fetus through vertical transmission from mother to child.

During fetal brain development, genetic defects, environmental disturbances or pathogens can lead to defective mitosis or apoptosis of neural stem cells, which can disrupt the stem cell homeostasis and affect the differentiation of stem cells to other neural cells, manifesting as neurodevelopmental disorders, such as microcephaly and multiple sclerosis [34]. And the formation of neural circuits and changes in their function can lead to other mental disorders, such as ASD and schizophrenia [31]. It was found that SARS-CoV-2 may invade the CNS through blood circulation and peripheral nerve via the olfactory nerve [35].

The immune system plays an important role in nervous system injury caused by viral infection [36]. Persistent infection with SARS-CoV-2 and its infection of macrophages, microglia, and astrocytes in the CNS activates the inflammatory response of the body, causing immune system damage and brain injury [37]. At the same time, immune system damage and peripheral lymphocytopenia caused by SARS-CoV-2 increase the risk of secondary bacterial infections and exacerbate neurological damage [38]. A large number of deaths caused by COVID-19, most of which are due to multiple organ failures caused by viral-induced systemic inflammatory response syndrome (SIRS) or SIRS-like immune disorders [39].

Angiotensin-converting enzyme 2 (ACE2), a cardiovascular and cerebrovascular protective factor, is also abundantly expressed in glial cells, and SARS-CoV-2 has a high affinity for ACE2, and its spine protein interacts with ACE2 to disrupt the BBB and attack the nervous system. Children complete myelin development by the age of 2 years, and SARS-CoV-2 infection of oligodendrocytes causes demyelinating lesions, such as multiple sclerosis [40]. Patients with COVID-19 often present with severe hypoxia, and that hypoxia injury in the fetus can cause secondary damage to the nervous system [41].

### Increased mental stress in the general environment

The form of COVID-19 remains severe and people are inevitably affected and prone to stress and anxiety, and depression [42]. A meta-analysis study enrolling 27475 subjects showed ~25% of anxiety and 28% of depression in patients with COVID-19 [43]. Another cross-national study during the COVID-19 pandemic showed that 43% of the 6894 pregnant women and mothers felt elevated stress, 31% felt anxious or depressed, and 53% felt lonely [44]. It is suggested that pregnant women, as a more vulnerable group, are more emotionally vulnerable during their pregnancy and suffer from higher levels of psychological stress. In turn, excessive maternal stress during pregnancy produces prenatal stress damage that increases the risk of fetal neurodevelopmental disorders [45]. Animal studies have shown that prenatal stress leads to shorter and less complex dendrites, reduced myelin, and altered synapses [46]. To determine whether changes in brain function occur in the offspring of women with high maternal stress, Thomason et al. performed functional magnetic resonance imaging on 118 fetuses at a mean gestational age of 32.9 weeks and found that increased maternal prenatal stress and negative emotions were associated with alterations in fetal frontoparietal, striatal, and temporoparietal neural connectivity (*β* = 0.82, *P* < 0.001), suggesting that high maternal stress and negative emotions during the pregnancy can have an unwanted impact on the nervous system of the offspring [47].

**Fig. 1:**
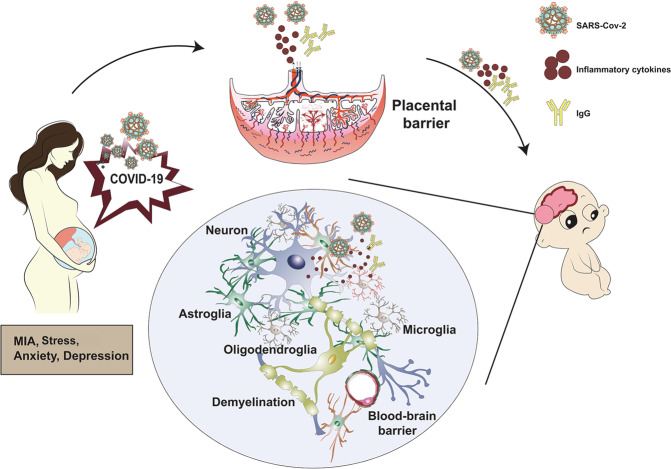
Potential mechanisms of SARS-Cov-2 infection on the nervous system in the offspring during the pregnancy. MIA maternal immune activation.

Deoni et al. found that children born after the COVID-19 pandemic were found to have lower developmental scores and these children had significantly lower language, motor, and overall cognitive skills [48]. Anti-epidemic measures may block parent-child emotional interactions and negatively affect children’s brains and behavior [49]. What’s more, this may be related to maternal stress and negative emotions during pregnancy affecting the structure and connectivity of the developing brain of the fetus, leading to potential delays in its motor, cognitive, and behavioral development [50]. The fetal brain is born with a strong capacity to learn and adapt, but is also very fragile and vulnerable to environmental exposures [51]. Maternal-fetal interaction and “kangaroo” care facilitate neurodevelopmental processes, including myelin formation and synaptogenesis [52]. On the other hand, exposure of the fetus to stress-related hormones such as cortisol might affect the structural and functional changes of the brain [53]. This is likely that higher cortisol levels in the mother during the pregnancy lead to lower cortisol levels in the infant at birth, which can dysregulate the hypothalamic-pituitary-adrenal axis and affect neurological development in the newborn [54] (Fig. 1).

## Prophylactic and treatment strategies

**Fig. 2:**
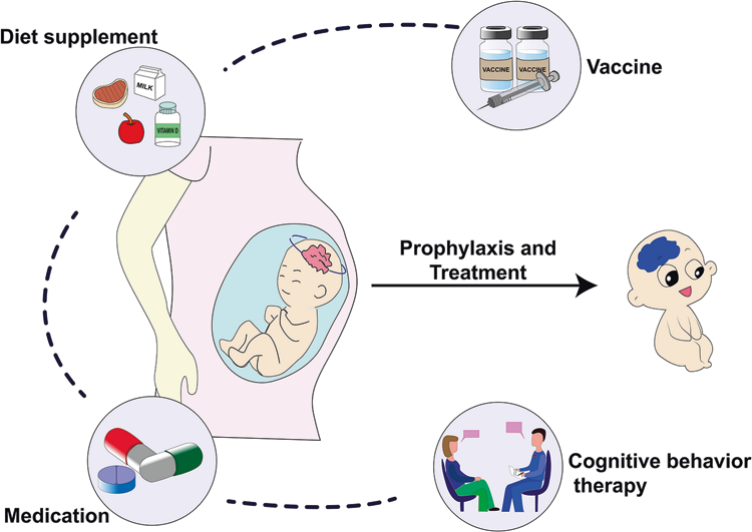
Prophylactic and treatment strategies. Effective prophylactic and treatment strategies for potential risks of offspring’s neuropsychiatric disorders in pregnant women during the COVID-19 pandemic.

There is still no specific medicine for COVID-19, and the most common treatments for infection in pregnant women are antibiotics, antivirals, and oxygen support. However, both the public and the scientific community pay great attention to the safety and impacts of these treatment measures on the fetus. A meta-study analysis showed that the use of antibiotics and antivirals is higher in Asian countries than in other countries, suggesting a possibility of antibiotic abuse [55]. The use of immunosuppressive drugs is associated with the occurrence of adverse outcomes and potentially serious complications, and their use should be minimized, and improved therapeutic approaches through risk stratification are recommended. Therefore, effective prophylactic is important for disease management in pregnant women. Given that current studies have not reported serious adverse reactions in pregnant women after receiving the COVID-19 vaccine [56], a vaccine with nutritional support seems to be an effective approach. For pregnant women who have already developed psychological problems, appropriate use of drug medication and psychological treatment are recommended and prescribed under the guidance of physicians (Fig. 2).

### Vaccination

Vaccination against infectious pathogens is one of the most influential public health interventions to reduce infection-related morbidity and mortality worldwide [57]. Theoretically, anti-SARS-CoV-2 immunoglobulins (IgG and IgA) are transmitted to the newborn through the placenta and breast milk after vaccination of pregnant women, providing humoral immunity. Regarding the safety of the vaccine in pregnant women, several studies have shown that no serious adverse events occurred in pregnant and lactating women who received the Pfizer/BioNTech vaccine, and no vaccine-associated mRNA was detected in breast milk collected 4 to 48 hours after vaccination, indicating that the vaccine is safe, although more studies are needed to analyze its effectiveness and impact on the offspring in the future [58–60]. Societies such as the American College of Obstetricians and Gynecologists (ACOG) and the Society for Maternal-Fetal Medicine (SMFM) continue to advocate for the availability of COVID-19 vaccine to pregnant and breastfeeding women [61]. WHO revised its statement on January 29, 2021, to allow vaccination of pregnant women at high risk of SARS-CoV-2 exposure (e.g., health workers) or with comorbidities that increase their risk of serious illness after consultation with their health care providers [56].

### Nutrition

Based on previous experience with disease pandemics and experience with other respiratory viruses and animal models, increasing maternal choline levels and other nutrient levels may reduce the effects of infection on fetal brain development [25]. Choline supplementation may reduce the increase in fetal IL-6 levels caused by RNA virus stimulation and may decrease anxiety [62]. Maternal choline levels are associated with the development of attention and orienting regulation in early childhood [63]. Maternal vitamin D deficiency was found to lead to altered placental pathology and increased risk of bacterial vaginitis, but excessive vitamin D levels were associated with increased IL-6 levels [64, 65]. Therefore, it is recommended that pregnant women consume vitamin D with folic acid according to a standard prenatal vitamin formula and moderate choline supplementation from beef, egg yolk, and soy.

### Antidepressants

The use of antidepressants such as selective serotonin reuptake inhibitor (SSRI) may be associated with a reduced risk of clinical deterioration in patients with SARS-CoV-2 infection. It may benefit depression patients with COVID-19 infection, since SSRIs exert anti-inflammatory effects on the damaged striatal neurons [28, 66]. Although using antidepressants during the pregnancy may increase the risk of ASD in offspring [67], a subsequent systematic review did not show an absolute contraindication to antidepressants and there was insufficient evidence of an association between antidepressants and adverse events [68]. Therefore, considering the safety of medication use in pregnant women, clinicians should balance the risks and benefits to develop an optimal treatment strategy on the basis of the actual situation.

### Psychological intervention

Psychotherapy is a safer strategy with less adverse effects than pharmacological treatments. Cognitive behavior therapy (CBT) is currently the most well-studied and popular treatment modality [69]. In view of the fact that maternal stress and hair cortisol levels are associated with motor and cognitive neurodevelopment of the fetus at 6 months of age [70]. A recent randomized controlled trial showed that CBT reduced cortisol levels in the hair of pregnant women and improved psychological stress and psychiatric symptoms [71]. The decreased cortisol levels in the hair are beneficial to the physical and mental health of pregnant women and their fetuses, so the use of CBT for stress and negative emotions could be greatly encouraged.

## Conclusion

COVID-19 affects fetal neurological development through multiple pathways during the pregnancy, although most newborns born in a COVID-19 pandemic setting are not directly infected with SARS-CoV-2. We need to pay attention not only to the neurological symptoms of neonatal impairment at birth, but also to the neuropsychiatric symptoms during the growth, and therefore improving the prognosis by early intervention. There is still a need for extensive follow-up studies to determine whether the fetal damage will be continued in adulthood, and new-onset psychiatric symptoms will be developed in adulthood. Most importantly, it is essential to create a safer environment and provide great support for pregnant women and their offspring.
